# Discovery of ETI41 and ETI60: novel selective endosomal Toll-like receptor inhibitors for the treatment of autoimmune diseases

**DOI:** 10.1038/s12276-025-01526-w

**Published:** 2025-09-01

**Authors:** Uisuk Jeong, Wang Hee Lee, Yang Seon Choi, Muhammad Haseeb, Wook-Young Baek, Ji Hye Han, Hongjoon Choi, Moon Suk Kim, Chang-Hee Suh, Wook Kim, Sangdun Choi

**Affiliations:** 1 S&K Therapeutics, Suwon, Korea; 2 Department of Molecular Science and Technology, Ajou University, Suwon, Korea; 3 Advanced College of Bio-convergence Engineering, Ajou University, Suwon, Korea

**Keywords:** Systemic lupus erythematosus, Psoriasis

## Abstract

Endosomal Toll-like receptors (TLRs, including TLR3, TLR7, TLR8 and TLR9) play crucial roles in immune responses by recognizing pathogen-associated molecular patterns; however, their aberrant activation is implicated in inflammatory and autoimmune diseases. Developing endosomal TLR inhibitors against autoimmune diseases is clinically essential. Here we synthesized and optimized a series of compounds based on a candidate structure. The lead compounds, ETI41 and ETI60, potently inhibited endosomal TLR-mediated pro-inflammatory signaling with nanomolar activity in cellular, biophysical and in vivo assays. Both ETI41 and ETI60 selectively inhibited endosomal TLRs without affecting surface TLRs, as confirmed by immunoblotting and biophysical analyses. RNA sequencing revealed that these inhibitors modulated the expression of genes associated with inflammation. In vivo studies have shown that oral administration of ETI41 or ETI60 effectively ameliorates symptoms in mouse models of psoriasis, and systemic lupus erythematosus. These findings indicate that ETI41 and ETI60 hold significant potential as therapeutic agents for the treatment of autoimmune and inflammatory diseases through selective targeting of endosomal TLRs.

## Targeting endosomal TLRs to treat autoimmune diseases

Toll-like receptors (TLRs) are crucial for our immune system, helping to detect harmful invaders such as bacteria and viruses. This study aimed to find new treatments by targeting these receptors. Researchers developed two new compounds, ETI41 and ETI60, designed to inhibit specific TLRs. They tested these compounds in laboratory-grown cells and mouse models of psoriasis and lupus. The compounds were found to reduce inflammation without being toxic to cells. They worked by blocking the signals that lead to inflammation. In experiments, ETI41 and ETI60 effectively reduced symptoms in mice with psoriasis and lupus. The compounds decreased skin inflammation and improved disease markers in blood tests. These findings suggest that ETI41 and ETI60 could be promising treatments for autoimmune diseases.

This summary was initially drafted using artificial intelligence, then revised and fact-checked by the author.

## Introduction

Toll-like receptors (TLRs) are pattern-recognition receptors that play a pivotal role in initiating the immune responses of the host to pathogen- and damage-associated molecular patterns (PAMPs and DAMPs). These receptors recognize a broad spectrum of pathogens, including bacteria, viruses, fungi and parasites, thereby orchestrating the host defense mechanisms^1^. Upon recognition of PAMPs and DAMPs, TLRs recruit adaptor proteins, which in turn trigger pro-inflammatory signaling pathways. This leads to the activation of nuclear factor kappa-light-chain-enhancer of activated B cells (NF-κB) and immature reticulocyte fraction (IRF) and the subsequent upregulation of inflammatory cytokines, chemokines and interferons (IFNs)^2,3^. TLRs are integral to both innate and adaptive immune responses, maintaining immune homeostasis and defending against pathogens. However, dysregulated TLR signaling has been implicated in various autoimmune and inflammatory diseases, including psoriasis, systemic lupus erythematosus (SLE), rheumatoid arthritis, type 1 diabetes and multiple sclerosis^4,5^. Genetic variations in TLR pathways, such as TLR7 and TLR9 in SLE or TLR4 in rheumatoid arthritis, contribute to aberrant immune activation, leading to chronic inflammation and tissue damage^6–8^. These associations highlight the need for targeted TLR inhibitors as potential therapeutic strategies for autoimmune diseases.

The human TLR family comprises ten distinct receptors, designated as TLR1 through TLR10. Plasma membrane TLRs include TLR1, TLR2, TLR4, TLR5, TLR6 and TLR10, whereas endosomal TLRs, including TLR3, TLR7, TLR8 and TLR9, are localized within the endosomal compartments of immune cells^9,10^. Structurally, these endosomal TLRs share a type I transmembrane topology, featuring extracellular leucine-rich repeat domains for ligand recognition and intracellular Toll/interleukin-1 receptor domains for downstream signaling^11,12^. Despite these structural similarities, each TLR exhibits unique ligand specificity.

TLRs 7–9 possess two distinct ligand-binding sites. Site 1 recognizes small molecules, such as imiquimod (IMQ) and resiquimod (R848) for TLR7 and TLR8, respectively, while a TCG motif oligodeoxynucleotide (ODN) with a 5′-xCx DNA is recognized by TLR9^13–15^. Site 2 binds to single-stranded RNA for TLR7 and TLR8, and unmethylated CpG DNA motifs for TLR9^16^. Activation of these endosomal TLRs initiates a signaling cascade involving adaptor proteins, such as MyD88 and TRIF, leading to the activation of transcription factors, such as NF-κB, AP-1, mitogen-activated protein kinase (MAPK) and IRF3/7^3^. Excessive activation of endosomal TLRs notably contributes to the pathogenesis of various inflammatory and autoimmune diseases^17,18^. Furthermore, targeting multiple endosomal TLRs provides a broad-spectrum therapeutic approach, especially in diseases where multiple TLR pathways contribute to disease progression. Thus, endosomal TLRs have emerged as promising therapeutic targets. However, only a limited number of small-molecule inhibitors, such as enpatoran (M5049) and MHV370, have progressed to clinical trials^19,20^.

In this study, we developed endosomal TLR inhibitors through structure–activity relationship (SAR)-based modification and optimization. This approach led to the discovery of highly potent and selective inhibitors of TLR7 and TLR9. These inhibitors effectively suppressed the production of inflammatory cytokines in multiple cell lines and primary cells responsive to TLRs, while exhibiting negligible cytotoxicity. Target-specific binding was validated through immunoblotting and biophysical analyses. Furthermore, in vivo studies have demonstrated the therapeutic efficacy of two compounds, ETI41 (endosomal TLR inhibitor 41) and ETI60, in models of psoriasis, and SLE. Oral administration of these compounds provides substantial protection against autoimmune diseases, highlighting their potential as promising therapeutic agents.

## Materials and methods

### Compound synthesis

ETI41 and ETI60 were synthesized at WuXi AppTec, China, as explained in detail in the Supplementary Information.

### Cell lines and reagents

The RAW 264.7 cell line (Korean Cell Line Bank) was cultured in Dulbecco’s modified Eagle medium with high glucose (HyClone Laboratories). THP-1 cells (ATCC) were cultured in Roswell Park Memorial Institute (RPMI) 1640 medium (HyClone Laboratories) and differentiated into M0 macrophages using 16 nM phorbol 12-myristate 13-acetate (Sigma-Aldrich) for 48 h. The supernatant was replaced with fresh medium 18 h before treatment. The Daudi cell line (Korean Cell Line Bank) was cultured in RPMI 1640. All culture media were supplemented with 1% penicillin–streptomycin (HyClone Laboratories) and 10% fetal bovine serum (Thermo Fisher Scientific). All cells were maintained at 37 °C in a humidified atmosphere containing 5% CO_2_, and fresh medium was added daily.

TLR agonists, including Pam3CSK4 (TLR1/TLR2), FSL-1 (TLR2/TLR6), Poly I:C (TLR3), IMQ (TLR7), ORN06/LyoVec (mouse TLR7), TL8-506 (TLR8) and R848 (TLR7/8) were purchased from Invivogen. Lipopolysaccharide (LPS) from *Escherichia coli* O111:B4 (a TLR4 agonist) was obtained from Sigma-Aldrich. To induce TLR9-specific stimulation, ODN2395 (Class C ODN with unmethylated CpG, 5′-TCG TTT TCG GCG CGC GCC G-3′) was synthesized by Bioneer with complete phosphorothioate backbone modification. All TLR agonists were dissolved in deionized water and added to the culture medium to ensure TLR activity.

### MTT assay

RAW 264.7 and THP-1 (M0 type) cells were seeded into 96-well plates at a density of 2 × 10^4^ cells per well and incubated overnight. Wells were treated with the test compounds or a dimethyl sulfoxide (DMSO) negative control (DMSO; Biosesang) at a final concentration of 0.25%. After 24 h, the supernatant was removed, and 500 μg/ml of 3-[4,5-dimethylthiazol-2-yl]-2,5 diphenyl tetrazolium bromide (MTT) solution (Invivogen) was added. After a 3 h incubation, the supernatant was removed, and DMSO was added to dissolve the formazan dye. The optical absorbance was measured at 595 nm using a BioTek Synergy HTX multimode microplate reader (BioTek Instruments) and normalized to that of the negative control.

### Water-soluble tetrazolium assay

Daudi cells were seeded into 96-well plates at a density of 5 × 10^4^ cells per well. After 24 h of treatment with the test compounds, 1/10 of the supernatant was removed and replaced with Cyto X solution (LPS Solution). The optical absorbance was measured at 450 nm using a BioTek Synergy HTX multimode microplate reader, and values were normalized to those of the negative control.

### Enzyme-linked immunosorbent assay (ELISA)

RAW 264.7 and differentiated THP-1 cells were seeded into 96-well plates at 2 × 10^4^ cells per well, while Daudi cells were seeded at 5 × 10^4^ cells per well. After stabilization for 30 min, cells were stimulated with specific TLR agonists for 4 or 24 h. Supernatants were diluted and transferred to precoated ELISA plates to measure TNF-α secretion using kits from Invitrogen. Optical absorbance was measured using a BioTek Synergy HTX multimode microplate reader, and data were interpolated using BioTek Gen5 software.

### Western blot analysis

RAW 264.7 cells were seeded into 60-mm dishes at 1 × 10^6^ cells per dish. After 30 min of treatment with ETI41 or ETI60, the cells were stimulated with 2 μM IMQ (a TLR7 agonist) or 1 μM ODN2395 (a TLR9 agonist). Lysates were prepared using a Mammalian Protein Extraction Kit (Thermo Fisher Scientific) with protease and phosphatase inhibitors. Nuclear proteins were extracted using an NE-PER Nuclear and Cytoplasmic Extraction Kit (Thermo Fisher Scientific). Proteins were quantified using a bicinchoninic acid kit (Sigma-Aldrich), separated by SDS–PAGE and transferred onto nitrocellulose membranes (GE Healthcare). Membranes were incubated with specific primary antibodies and horseradish peroxidase-conjugated secondary antibodies. Phospho-SAPK/JNK (9251S, lot no. 27), phospho-p38 MAPK (9211S, lot no. 26), IκB-α (9242S, lot no. 11), ERK 1/2 (9102S, lot no. 27), IRF-7 (72073S, lot no. 1), NF-κB p65 (6956S, lot no. 10), SAPK/JNK (9252S, lot no. 18), p38 MAPK (9212S, lot no. 27) are purchased from Cell Signaling Technology Inc. Phospho-ERK 1/2 (sc-81492, lot no. 1123), β-actin (sc47778, lot no. H1924) and Lamin A/C (sc-20681, lot no. 131913) are purchased from Santa Cruz Biotechnology. Protein bands were detected using a chemiluminescent substrate (SuperSignal West Pico PLUS; Thermo Fisher Scientific) and visualized using a Fuji LAS-3000 system (Fujifilm).

### Real-time quantitative PCR

Bone marrow cells from 8-week-old C57BL/6 mice (Jackson Laboratory) were cultured in RPMI 1640 with 20 ng/ml granulocyte-macrophage colony-stimulating factor (GM-CSF; Thermo Fisher Scientific) for 8 days. Bone marrow-derived dendritic cell (BMDC) purity was confirmed by fluorescence-activated cell sorting (FACS), showing 84% F4/80^−^ and CD11c^+^ cells. BMDCs were seeded into six-well plates at a density of 2 × 10^5^ cells per well; they treated with ETI41 for 30 min and then with ODN2395 for 2 h. Total mRNA was extracted using TRIzol reagent (Thermo Fisher Scientific) and reverse transcribed using the ReverTra Ace qPCR RT Kit (Toyobo). mRNA levels were determined using SYBR Green PCR Master Mix (Kapa Biosystems) and a CFX Connect RT-PCR System (Bio-Rad). Primers used were directed against IL-12p40, IFN-β, CD40 and GAPDH. Data were normalized to GAPDH expression: IL-12p40 (F; 5′-GCT CAG GAT CGC TAT TAC AAT TCC-3′, R; 5′-TCT TCC TTA ATG TCT TCC ACT TTT CTT-3′), IFN-β (F; 5′-CGT GGG AGA TGT CCT CAA CT-3′, R; 5′-AGA TCT CTG CTC GGA CCA CC-3′), CD40 (F; 5′-TCT AGA GTC CCG GAT GCG AG-3′, R; 5′-GGA TCC TCA AGG CTA TGC TGT CG-3′) and GAPDH (F; 5′-CAT CAC TGC CAC CCA GAA GAC T-3′, R; 5′-CCA GTG AGC TTC CCG TTC A-3′).

### QuantSeq 3′ mRNA sequencing analysis

RNA quality was assessed using a TapeStation 4000 system (Agilent Technologies). Libraries were prepared using a QuantSeq 3 mRNA-Seq Library Prep Kit FWD (Lexogen) and sequenced using a NextSeq 550 System (Illumina). Sequences were trimmed using BBDuk and aligned with Bowtie2, and the reads were counted using Bedtools^21–23^. Data were normalized using edgeR software (Ebiogen) and analyzed for gene function using the Database for Annotation, Visualization, and Integrated Discovery) and for pathway analysis using KEGG Mapper^24,25^. RNA-seq data were deposited in the Gene Expression Omnibus database under accession number GSE255890.

### SPR analysis

Surface plasmon resonance (SPR) analysis was conducted using an SR7500DC SPR System (Reichert) with a CMDH chip for human TLR7 and murine TLR9 (R&D Systems) and a Biacore T200 platform (Cytiva) with a CM5 chip for TLR9–ODN binding. The running buffer contained 0.5% DMSO and phosphate-buffered saline, and 50 mM NaOH was used for regeneration. ETI series compounds were serially diluted from 500 μM and tested for binding affinity. Binding curves were analyzed using Scrubber2 and Biacore T200 evaluation softwares. The dissociation constants (*K*_D_) were calculated as *K*_D_ = *k*_d_/*k*_a_.

### FACS analysis

RAW 264.7 cells were seeded into 60-mm dishes at 2 × 10^6^ cells per dish. After 30 min of treatment with ETI41 or ETI60, cells were incubated with 1 μM of 3’-FAM-labeled ODN 2395 (Bioneer Inc.) for 30 min. Fluorescence intensity was measured using a NovoCyte Flow Cytometer (Agilent Technologies) and analyzed using NovoExpress software.

### pH assay

RAW 264.7 cells were seeded into black 96-well plates at 5 × 10^6^ cells per well and incubated overnight. After washing, the cells were treated with phenol-free RPMI 1640 medium (Gibco). Following 30 min of treatment with ETI41 or ETI60, cells were incubated with 20 µg/ml of pHrodo Red Dextran (Thermo Fisher Scientific) or 10 µM LysoSensor Yellow/Blue DND-160 (Thermo Fisher Scientific). Fluorescence was measured using a BioTek Synergy HTX Multimode Microplate Reader. Data were analyzed using the BioTek Gen5 software.

### Induction of psoriasis in a murine model

Six-week-old female C57BL/6J mice were assigned to the following treatment groups: vehicle (*n* = 5), IMQ (*n* = 5), ETI41 (*n* = 4) and ETI60 (*n* = 5). The mice were obtained from Orient Bio to evaluate the effects of ETI41 and ETI60 on induced psoriasis-like symptoms. This study was approved by the Institutional Animal Care and Use Committee (IACUC; approval no. 2014-0007). Psoriasis was induced using Aldara cream (3M Pharmaceuticals), which was applied topically at 62.5 mg/cm² daily from the day after hair removal until the fourth day. Mice were orally administered 60 mg/kg ETI41 or ETI60 daily commencing on the second day of induction. For IL-23-induced psoriasis, the mice were assigned to the following treatment groups: vehicle (*n* = 5), IL-23 (*n* = 5), ETI41 (*n* = 5), ETI60 (*n* = 5) and anti-IL-17 Ab (*n* = 5). Recombinant mouse IL-23 (0.25 mg/kg; BioLegend) was administered via intradermal injection around the ear daily, starting the day after hair removal and continuing until the eighth day. Positive control mice received intraperitoneal (IP) injections of 30 mg/kg anti-mouse IL-17A antibody (BioLegend) on days 2, 5 and 8, while test group mice were administered daily oral doses of 60 mg/kg of ETI41 or ETI60. Changes in body weight and Psoriasis Area and Severity Index (PASI) scores (erythema, scaling and thickness) were recorded daily. Mice were initially anesthetized in an induction chamber with a gas mixture of 4% isoflurane in 70:30 NO_2_ and O_2_, delivered at a flow rate of 0.8 l/min. Subsequently, the isoflurane concentration was reduced to 2%, and the mice were euthanized under anesthesia via endotracheal intubation. Skin samples were collected, fixed and stained with hematoxylin and eosin for histological analysis. Dermal and epidermal thicknesses were measured using a Leica DMi8 microscope (Leica). Immunohistochemical analyses were performed using primary antibodies against CD68 (R&D Systems, MAB101141), Ki-67 (Cell Signaling Technology, 12202), IL-17A (Novus Biologicals, NBP1-72027) and IL-23 (Novus Biologicals, NBP1-76697), followed by detection using biotinylated secondary antibodies (Vector Laboratories) and diaminobenzidine (Vector Laboratories) visualization. Fluorescence measurements for IL-23-induced psoriasis were performed using primary antibodies against CD68 and Ki-67, followed by Alexa Fluor-conjugated secondary antibodies (Life Technologies). Fluorescence was analyzed using a Leica DMi8 microscope.

### Murine SLE model

MRL/MpJ-Fas^lpr^/J lupus-prone mice were purchased from Jackson Laboratory to evaluate the effects of ETI41 and ETI60 on the pathophysiology of genetic SLE. This study was approved by the IACUC, Ajou University (approval no. 2019-0046). Fourteen-week-old mice were assigned to the following treatment groups: vehicle (*n* = 3), ETI60 (*n* = 3), ETI41 (*n* = 4) and HCQ (*n* = 3). These groups were administered daily oral doses of 30 mg/kg ETI41 or ETI60, or 60 mg/kg HCQ for 39 days. All compounds were dissolved in a vehicle comprising ethanol, polyethylene glycol (PEG400) and distilled water at a 1:4:5 ratio. The body weights of the mice were measured every 3 days. Mice were anesthetized in an induction chamber with a gas mixture of 4% isoflurane in 70:30 NO_2_ and O_2_, delivered at a flow rate of 0.8 l/min. Upon completion of the treatment period, paired axillary and popliteal lymph nodes were collected and weighed. Blood samples were taken and maintained in BD Microtainer SST tubes (BD Biosciences), with serum isolated through centrifugation at 3,393*g* for 30 min at 4 °C. Serum markers, including complement component C3, ANA, anti-dsDNA and total IgG, were evaluated using ELISA kits (MyBioSource). Renal IgG levels were monitored by western blotting using anti-mouse IgG (Thermo Fisher Scientific) and β-actin antibodies (Bethyl Laboratories). Statistical significance among groups was analyzed using IBM SPSS Statistics (v.25.0; IBM Corporation), with statistical significance set at *P* < 0.05.

### Molecular docking

The cryo-electron microscopy structure of human TLR7 (PDB ID: 7CYN)^26^ was obtained from the Protein Data Bank^24,27^. A human TLR9 model was constructed through homology modeling using 3WPC as a template on the SWISS-MODEL web server^28,29^. The resulting model was protonated at pH 7.0, and the energy was minimized using Amber12: Extended Hückel Theory force field in Molecular Operating Environment software^30^. Ligand-binding sites (Site I) were defined by ligands found in the crystal structures of TLR7 and 9^13,15^. ETI41 and ETI60 were docked at Site I using the MMFF94x force field and the London dG scoring system. Thirty conformations were generated for each compound, and the best hits were selected on the basis of their highest docking (binding affinity) scores. Ligand interactions were evaluated using the Biovia Discovery Studio Visualizer^31^.

### Statistical methods

One-tailed paired Student’s *t*-tests were performed using Microsoft Excel (Microsoft). Semi-logarithmic conversions and nonregression curve analyses for calculating 50% lethal concentration (LC_50_) and 50% inhibitory concentration (IC_50_) values were conducted using GraphPad Prism 7.0 (GraphPad). The therapeutic index was calculated as IC_50_/LC_50_. Animal experimental results are expressed as mean ± standard deviation and analyzed using SPSS v.20 (IBM SPSS Statistics). Homogeneity of variance was assessed using Levene’s test.

## Results

### Identification of potent endosomal TLR inhibitors

**Fig. 1:**
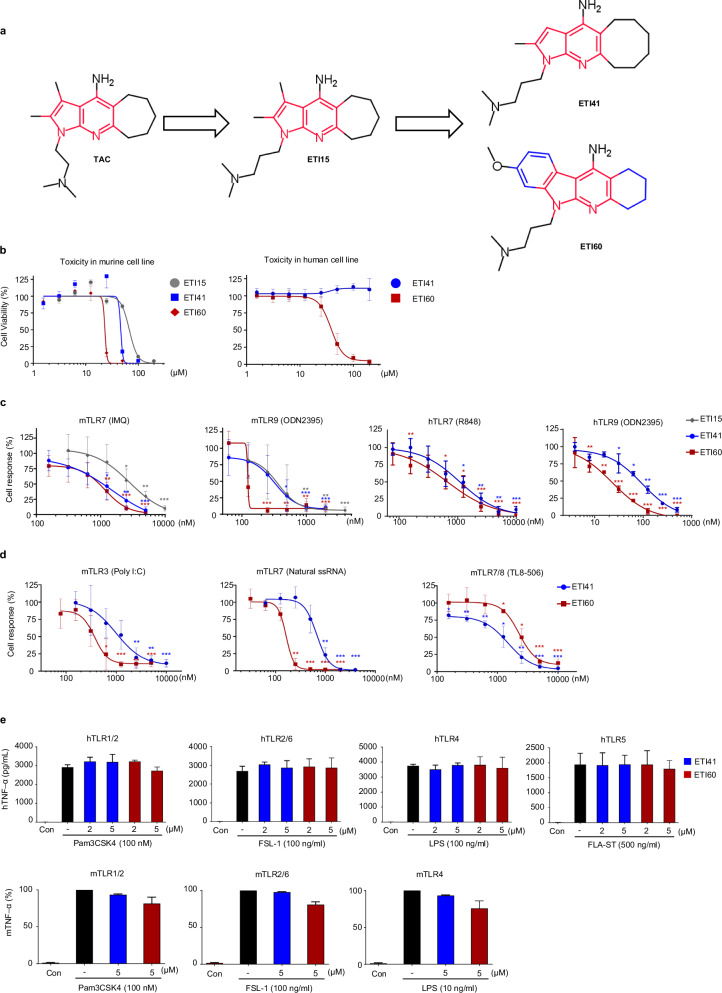
ETI41 and ETI60 potently and selectively inhibit endosomal TLRs. **a** Chemical structures of the main scaffold, TAC (red), and the potent inhibitors, ETI41 and ETI60. **b** Cell survival curve according to ETI concentration (1.6–200 μM) was measured by MTT assay in murine RAW 264.7 cell line and water-soluble tetrazolium assay in a human Daudi cell line. **c** Inhibitory effects of ETI15, ETI41 and ETI60 (ranging from 3.9 nM to 10 μM) on TLR7 and TLR9 were assessed by quantifying TNF-α secretion in murine RAW 264.7 cells and human Daudi cells, respectively. **d** ETI41 and ETI60 inhibited TLR3, TLR7 and TLR8 in a concentration-dependent manner (31.2 nM to 10 μM), as indicated by the reduction in TNF-α secretion in mouse RAW 264.7 cells. **e** Specificities of ETI41 and ETI60 were confirmed by measuring TNF-α secretion in surface TLRs (TLR1, TLR2, TLR4, TLR5 and TLR6. The cells were activated with agonistic ligands: TLR1/2 (FSL-1, 100 ng/ml, 4 h), TLR2/6 (Pam3CSK4, 100 nM, 4 h), TLR3 (poly I:C, 2 μg/ml, 24 h), TLR4 (LPS, 10 or 100 ng/ml, 4 h), TLR5 (FLA-ST, 500 ng/ml, 4 h), TLR7 (ORN06/LyoVec, 2 μg/ml, 24 h; and IMQ, 1 μg/ml, 4 h), TLR8 (TL8-506, 2 μg/ml, 24 h) and TLR9 (ODN2395, 1 μM, 4 h) at various concentrations in RAW 264.7 cells, human Daudi cells and THP-1 cells. Data are from at least three independent experiments (*n* = 3) and statistical differences between the induced case and other cases were analyzed and verified using a one-tailed Student’s *t*-test (*P < 0.05, **P < 0.01, ***P < 0.001).

Previously, we identified a series of TLR-antagonistic compounds (TACs) using a quantitative SAR-based method^32^. Using 1,2,3-trimethyl-1*H*-pyrrolo[2,3-b]pyridin-4-amine as a starting point, we designed derivatives, with ETI15 (1-(3-(dimethylamino) propyl)-2,3-dimethyl-1,5,6,7,8,9-hexahydrocyclohepta[b]pyrrolo[3,2-e]pyridin-4-amine) emerging as a superior candidate with significant inhibitory activity against TLR7 and TLR9 (Fig. 1a). Through SAR-based optimization, we developed ETI41 by modifying the pyrrole group and replacing the 5*H*-cyclohepta[b]pyridine group with a 10-hexahydrocycloocta[b]pyridine moiety. Further optimization by incorporating 6-methoxyindoline into the pyrrole group and substituting the 5*H*-cyclohepta with a cyclohexane group led to the development of ETI60 (Fig. 1a; synthesis details are provided in the Supplementary Information).

### ETI41 and ETI60 inhibit endosomal TLR-mediated cytokine production

TLR activation triggers the NF-κB pathway, resulting in increased production of pro-inflammatory cytokines, such as TNF-α and IL-6. For detailed evaluation, the activity of TLRs for each cell line was optimized (data not shown) and the pharmacological activity of ETI41 and ETI60 was evaluated on the basis of toxicity and inhibitory effects in various cell lines. Stimulation with specific agonistic ligands for endosomal TLRs (poly I:C for TLR3, R848 and IMQ for TLR7, TL8-506 for TLR8 and ODN2395 for TLR9) significantly increased TNF-α production. ETI15 and its analogs, ETI41 and ETI60, effectively inhibited TNF-α production by mouse macrophages (RAW 264.7 cells) and human B lymphoblasts (Daudi cells) in a dose-dependent manner, without inducing cytotoxic effects (Fig. 1b–d and Supplementary Fig. 1). ETI41 exhibited inhibitory activity against endosomal TLRs with IC_50_ values ranging from approximately 100 to 1,000 nM, while ETI60 showed IC_50_ values from 20 to 300 nM for TLR3, TLR7 and TLR9, and approximately 2 µM for TLR8 (Tables 1 and 2).

**Table 1:** Therapeutic index of ETI15, ETI41 and ETI60 in RAW 264.7 cells.

RAW 264.7 cell line (unit: μM)					
Name	LC_50_	TLR7		TLR9	
		IC_50_ (mTNFα)	TI value	IC_50_ (mTNFα)	TI value
**ETI15**	67.9	2.81	24.16	0.36	241.50
**ETI41**	67.18	0.63	106.63	0.16	419.88
**ETI60**	51.19	0.68	75.65	0.12	422.71

TI therapeutic index, mTNFα mouse TNFα.

**Table 2:** Therapeutic values of ETI41 and ETI60 in various endosomal TLR conditions.

Cell line	Treatment	Target	Cytokine measurement	ETI41 (nM)	ETI60 (nM)
RAW 264.7	poly I:C	mTLR3	mTNFα	993.3	372.5
	TL8-506	mTLR8		1,489	2,272
Daudi	R848	hTLR7	hTNFα	946.8	667.5
	ODN2395	hTLR9		80.14	20.88
	Cell viability (LD_50_)		Cytotoxicity	>200,000	38,410

LD_50_ 50% lethal dose.

**Fig. 2:**
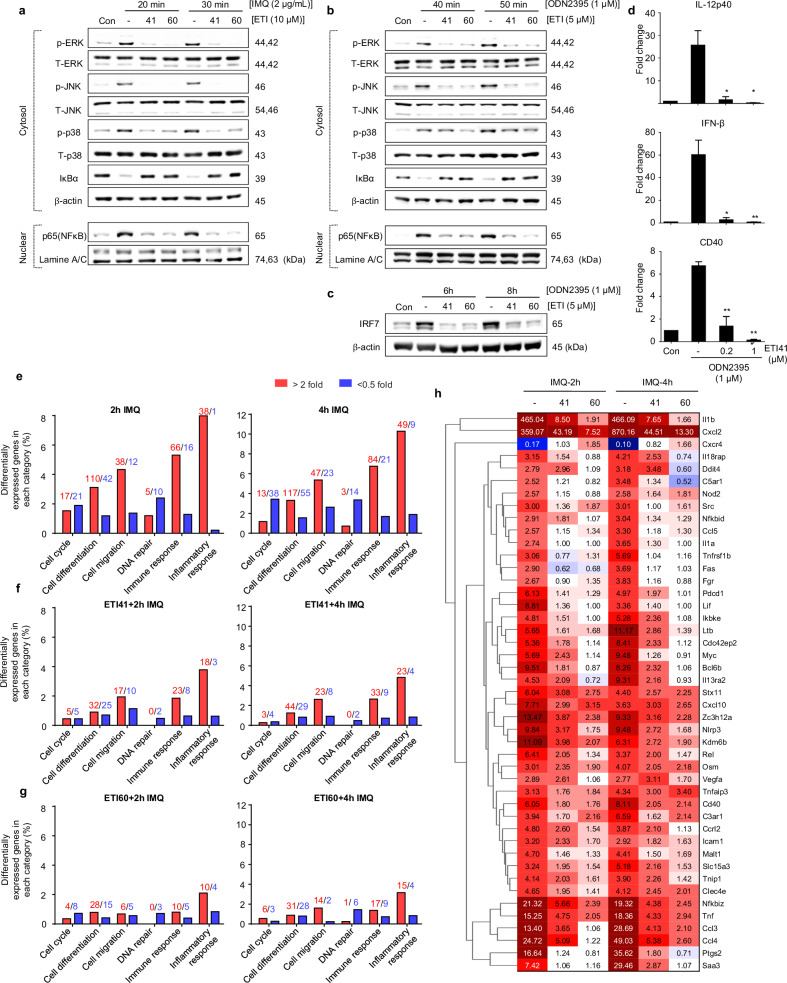
ETI41 and ETI60 inhibit TLR7- and TLR9-dependent downstream signaling. **a**, **b** Western blot analysis was used to quantify the protein levels of phosphorylated p-ERK, p-JNK, p38, Iκ-Bα and p65. RAW 264.7 cells were treated with 5 μM or 10 μM of ETI41 or ETI60, followed by exposure to IMQ (2 μg/ml) for 20 or 30 min (**a**) and ODN2395 (1 μM) for 40 or 50 min (**b**) respectively. β-Actin served as a loading control for the cytosol, while lamin A/C was utilized as a loading control for the nucleus. **c** Protein levels of IRF7 were measured after treatment with 5 μM of ETI41 or ETI60, followed by treatment with ODN2395 (1 μM) for 6 or 8 h. **d** BMDC cells from ex vivo were used to analyze mRNA expression levels of *IL-12p40*, *IFN-β* and *CD40*. Cells were pretreated with ETI41 (0.2 and 1 μM), followed by treatment with ODN2395 (1 μM) for 3 h. All experiments were independently conducted three times. Statistical differences between the induced and other cases were analyzed and verified using a one-tailed Student’s *t*-test (*P < 0.05, **P < 0.01, ***P < 0.001). **e**–**g** The TLR7 agonist IMQ (1 μM) was used in 3’ RNA-seq analysis to induce signaling associated with immune responses in RAW 264.7 cells. RNA-seq analysis showed that ETI41 and ETI60 at a concentration of 10 μM significantly changed gene expression patterns (fold change >2, *P* < 0.05) in various categories after 2 or 4 h of IMQ treatment. (**e**–**g**) depict changes in gene expression categories without pretreatment (**e**) after pretreatment with ETI41 (**f**) or after pretreatment with ETI60 (**g**) followed by IMQ stimulation. **h** Heatmaps depicting gene expression patterns within the inflammation and immune response categories (fold change >2.5, *P* < 0.05) of ETI41 or ETI60, dividing the time after IMQ treatment into 0, 2 and 4 h, respectively. Values of each fold change compared with the noninduced case are described, with upregulated genes shown in red and downregulated genes shown in blue.

Given the structural similarities among TLR family proteins, achieving high selectivity is challenging^33^. We tested the effects of ETI41 and ETI60 on various surface TLRs to determine their specificity. At concentrations of 2 and 5 μM, neither compound inhibited any plasma membrane TLR, including TLR1/TLR2, TLR2/TLR6, TLR4 and TLR5, in differentiated human THP-1 cells activated by FSL-1, Pam3CSK4, LPS and FLA-ST, respectively (Fig. 1e and Supplementary Fig. 1). TLRs activate transcription factors, including NF-κB, MAPKs, c-Jun N-terminal kinase (JNK), extracellular signal-regulated kinase (ERK) and IRF7, which regulate cellular functions such as proliferation and apoptosis. Western blot analysis confirmed that ETI41 and ETI60 inhibited IMQ- or ODN2395-induced phosphorylation of MAPKs (p-ERK, p-JNK and p-p38), nuclear translocation of the NF-κB p65 subunit, Iκ-Bα degradation and IRF7 expression in RAW 264.7 cells (Fig. 2a–c and Supplementary Figs. 2 and 3). Furthermore, ETI41 significantly inhibited the production of IL-12p40, IFN-β and CD40 in primary BMDCs stimulated with the TLR9 agonist ODN2395 (Fig. 2d).

### RNA-sequencing analysis demonstrates reprogramming of IMQ-induced inflammation by ETI41 and ETI60

RNA sequencing was used to identify changes in gene expression and elucidate the mechanisms of inhibition by ETI41 and ETI60. The TLR7 agonist IMQ was used to trigger signaling in RAW 264.7 murine macrophage cells, resulting in the expression of 23,282 genes. We categorized gene expression changes into six different types of signaling pathway, focusing on immune and inflammatory responses (Fig. 2e). The administration of ETI41 and ETI60 for 2 and 4 h induced changes in gene expression patterns compared with the control (Fig. 2f-g). ETI41 treatment resulted in 509 differentially expressed genes (DEGs), with 296 downregulated and 213 upregulated genes. ETI60 treatment identified 727 DEGs, including 380 downregulated and 347 upregulated genes. IMQ-induced cases showed higher expression of genes associated with inflammation, such as IL1-β, CXCL2, IL18RAP, TNF, PDCD1, NLRP3, NFKBIZ, CCL3, CCL4, KDM6B, ZC3H12A and PTGS2. ETI41 and ETI60 markedly reduced the expression of these genes in 2 and 4 h, respectively (Fig. 2h). Kyoto Encyclopedia of Genes and Genomes (KEGG) pathway analysis indicated that the identified DEGs were primarily involved in immune signaling pathways (Supplementary Fig. 4). In addition, we reevaluated 23,282 genes to assess potential off-target effects and identified DEGs, along with their associated pathways, following treatment with either ETI41 or ETI60 alone (Supplementary Fig. 5). These results suggested that ETI41 and ETI60 ameliorated IMQ-induced autoimmune diseases by suppressing the expression of inflammation-associated genes.

### Biophysical and computational analysis of ETI41 and ETI60 binding mechanisms

**Fig. 3:**
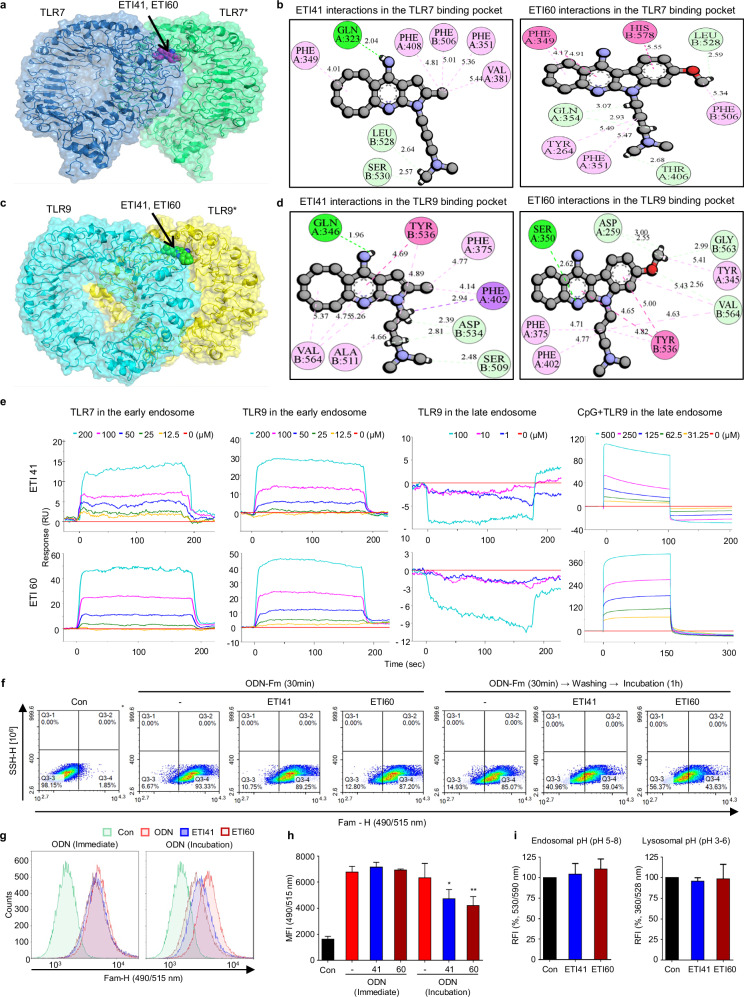
Binding mechanism of ETI41 and ETI60 with TLR7 and TLR9. **a** ETI41 and ETI60 bind to the extracellular domain of TLR7. **b** Detailed intermolecular interactions ofETI41 and ETI60 are shown with the key residues of TLR7. **c** ETI41 and ETI60 bind to the extracellular domain of TLR9. **d** Intermolecular interactions of ETI41 and ETI60 with the key residues of TLR9. The asterisk indicates chain A of TLR7 and TLR9. ETI41 and ETI60 are depicted in ball-and-stick models. Bonds are represented by lines, with distances measured in ångström (Å) units. Dark-green residues indicate conventional hydrogen bonds, light-green residues indicate carbon–hydrogen bonds, pink indicates pi–alkyl bonds, dark pink indicates pi–pi stacked bonds and purple indicates pi–sigma bonds. **e** SPR analysis of ETI41 and ETI60 binding to TLR7 and TLR9 in early and late endosomes at pH 7.4 and 5.5, respectively. **f**–**h** Panels illustrate the uptake and encapsulation of CpG-ODN in Daudi cells treated with ETI41 or ETI60, analyzed by flow cytometry: panel (**f**) presents representative dot plots, panel (**g**) shows overlay histograms of fluorescence intensity, and panel (**h**) quantifies the mean fluorescence intensity (MFI). **i** Effects of ETI41 and ETI60 were measured in endosomal and lysosomal acidic pH 5.5. Data presented are from three independent experiments (*n* = 3), and statistical differences between the induced case and other cases were analyzed and verified using a one-tailed Student’s *t*-test (*P < 0.05, **P < 0.01, ***P < 0.001).

Endosomal TLRs share structural similarities, including an N-terminal extracellular domain and leucine-rich repeats involved in TLR–ligand interactions^34^. Synthetic ligands such as IMQ, CL075 and R848 bind to Site I, corresponding to the nucleoside-binding site in TLR7 and TLR8^13,14^. This site overlaps with the 5′-xCx DNA motif of TLR9^15^. We used the cryo-electron microscopy structure of human TLR7 (PDB ID: 7CYN) and a homology model of human TLR9 for molecular docking^26^. ETI41 and ETI60 were docked onto Site I of TLR7 and 9, respectively, to elucidate their binding mechanisms (Fig. 3a,c). In TLR7, ETI41 and ETI60 exhibited strong interactions with key residues. The pyridine-amine moiety of ETI41 formed conventional hydrogen bonds with Q323, whereas the *N*,*N*-dimethylbutan-1-amine moiety interacted with L528 and S530. The cyclooctane moiety engaged in pi–alkyl interactions with F349, and the pyrrole moiety interacted with hydrophobic residues F408, F506, F351 and V381. ETI60 was surrounded by hydrophobic, polar uncharged and charged residues (Y264, F349, Q354, F351, T406, F506, L528 and H578), forming hydrogen and pi–alkyl bonds (Fig. 3b). For TLR9, both ETI41 and ETI60 demonstrated strong interactions with various residues, forming hydrogen, pi–alkyl and pi–pi stacked bonds (Fig. 3d). These interactions enhanced the stability of ETI41 and ETI60 within the binding pocket.

SPR confirmed the binding mechanisms of ETI41 and ETI60 with TLR7 and TLR9, respectively, under three conditions: (a) early endosome (pH 7.4), (b) late endosome without CpG (pH 5.5) and (c) late endosome with CpG (pH 5.5). (a) Both ETI41 and ETI60 were measured for parameters indicating binding affinity, including the *K*_D_ value presented in Table 3, and were further analyzed under conditions (b) and (c), depending on the presence or absence of ligand, to explain the binding mechanism occurring in TLR9. ETI41 and ETI60 showed similar binding patterns in all conditions. At neutral pH (that is, 7.4), a clear binding affinity was observed for both TLR7 and TLR9; however, binding was detected only in the late endosomal state where the natural ligand is present at acidic pH (that is, 5.5) (Fig. 3e). These findings suggested that ETI41 and ETI60 bind to Site I of TLR7 and TLR9, respectively.

**Table 3:** Binding affinity parameters (*k*_a_, *k*_d_ and *K*_D_) of ETI41 and ETI60 measured in TLR7 and TLR9.

Ligand	Analyte	*k*_a_ (1/Ms)	*k*_d_ (1/s)	*K*_D_ (M)
Fc-TLR7	ETI41	4 × 10^2^	0.21	5.40 × 10^-4^
	ETI60	20	0.124	6 × 10^-3^
Fc-TLR9	ETI41	1 × 10^1^	0.191	3 × 10^-3^
	ETI60	71	0.106	1.50 × 10^-3^

FACS analysis further validated the binding of ETI41 and ETI60. Unmethylated CpG ODNs, acting as ligands for TLR9, were fluorescently labeled, and their cellular penetration was monitored. After a 30-min treatment with unmethylated CpG ODNs, no changes were observed in the intracellular levels of CpG ODNs, ETI41 or ETI60, indicating that these inhibitors did not interfere with endocytosis. A reduction in fluorescence intensity was observed after washing, suggesting dissociation from Site I of TLR9 due to inhibition by ETI41 or ETI60 (Fig. 3f–h). This was in contrast to the decreased fluorescence intensity observed with the TLR9 antagonist ODN2088 during clathrin-dependent endocytosis (Supplementary Fig. 6). In addition, ETI41 and ETI60 did not significantly affect the pH of endosomes containing TLR3, TLR7, TLR8 and TLR9 (Fig. 3i).

### ETI41 and ETI60 ameliorate IMQ- and IL-23-induced psoriasis in mice

**Fig. 4:**
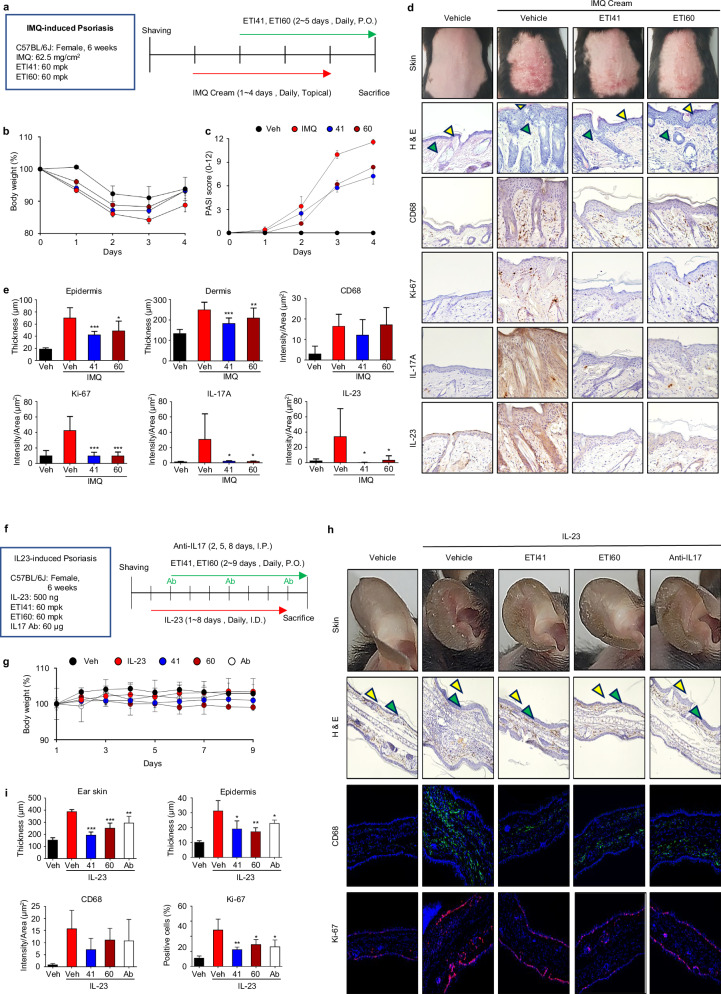
ETI41 and ETI60 ameliorate psoriasis induced by IMQ or IL-23 in mice. **a** Psoriasis was induced in female C57BL/6J mice by topically applying IMQ (62.5 mg/cm^2^). ETI41 and ETI60 were orally administered daily at a dose of 60 mg/kg. **b** Impact of ETI41 and ETI60 on body weight during treatment. **c** PASI score indicating disease severity. **d** Images of the back skin of mice were captured on the fifth day of treatment, revealing the therapeutic efficacy of ETI41 or ETI60 compared with the normal and untreated groups. Immunohistochemical staining of back skin sections from each group revealed the effects of ETI41 and ETI60 on thickness of the epidermis (yellow arrowhead) and dermis (green arrowhead). **e** The thickness of the epidermis and dermis, as well as the expression levels of CD68 (a macrophage marker), Ki-67, IL-17A and IL-23, were measured. **f** Psoriasis was induced in female C57BL/6J mice by injecting IL-23 (500 ng) into their ear skin. Anti-IL17A antibody (Ab, 60 µg) was administered by IP injection as a positive control on days 2, 5 and 8. ETI41 or ETI60 was administered orally at a daily dose of 60 mg/kg for 9 days. **g** Body weight was recorded during treatment. **h** On the ninth day of treatment, images of the mouse ears were acquired to demonstrate the therapeutic efficacy of ETI41 and ETI60 in comparison with the normal, vehicle or anti-IL17 groups. CD68 and Ki-67 expression patterns were determined in skin sections from a representative of each group using immunofluorescence. **i** Ear skin and epidermis thickness, CD68 expression and percentage of Ki-67 cells were examined from 4’,6-diamidino-2-phenylindole-stained cells. Statistical differences between the induced case and other cases were analyzed and verified using a one-tailed Student’s *t*-test (*P < 0.05, **P < 0.01, ***P < 0.001).

Endosomal TLRs play crucial roles in autoimmune diseases such as psoriasis^35,36^. We tested ETI41 and ETI60 in 6- to 7-week-old C57BL/6 mice with IMQ-induced psoriasis. Daily oral administration of 60 mg/kg significantly reduced the PASI scores, epidermal acanthosis, dermal thickness and keratinocyte proliferation. Treatment also inhibited IL-17A and IL-23 expression and dermal inflammatory cell infiltration (Fig. 4a–e). We investigated the efficacy of ETI41 and ETI60 treatment in an IL-23-induced murine model of psoriasis. IL-23 stimulated Th17 lymphocytes to secrete pro-inflammatory cytokines, such as IL-17, contributing to psoriasis development^37,38^.

Next, we investigated the therapeutic efficacy of ETI41 and ETI60 in an IL-23-induced mouse model of psoriasis. Psoriasis was induced through intradermal injections of recombinant murine IL-23. As a positive control, an IL-17A antibody was administered intraperitoneally at a dose of 30 mg/kg on days 2, 5 and 8 (Fig. 4f). ETI41 or ETI60 was orally administered daily at a dose of 60 mg/kg. Both compounds improved disease symptoms comparable to or better than the anti-IL-17A antibody, without significant differences in body weight (Fig. 4g). They significantly decreased ear and epidermal thicknesses, CD68 expression and Ki-67 keratinocyte proliferation (Fig 4h,i). These findings indicate that ETI41 and ETI60 effectively alleviate the progression of psoriasis, either through direct involvement of TLR7 or through an indirect involvement of the IL-23–Th17 axis.

### Therapeutic efficacy of ETI41 and ETI60 in an SLE mouse model

**Fig. 5:**
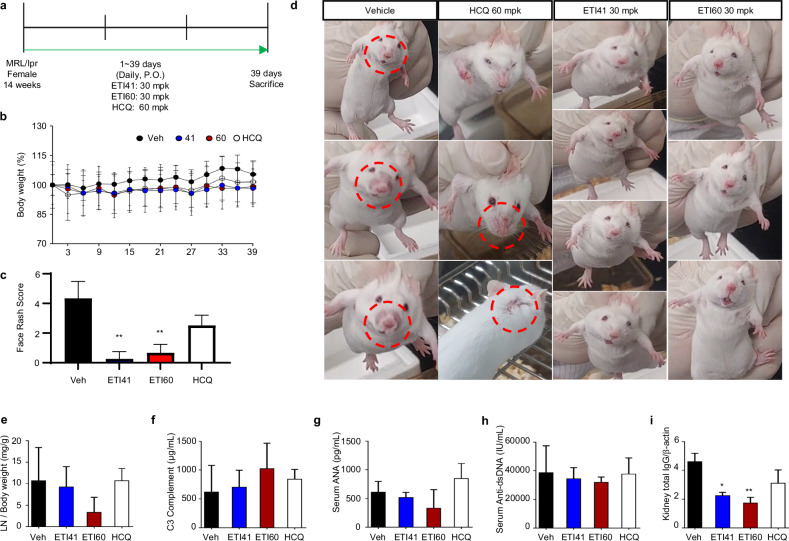
Therapeutic efficacy of ETI41 and ETI60 in SLE mouse model. **a** Summary of the experimental validation of ETI41 and ETI60 in the MRL/lpr female mouse model of SLE. ETI41 or ETI60 were orally administered at a dose of 30 mg/kg, and a positive control, HCQ, was administered at 60 mg/kg daily for 39 days. **b** Relative body weight was measured every third day from the first day until euthanasia. **c** Skin lesions were assessed on the final day using the following scoring criteria: 0, no visible rash; 1, mild redness without hair loss or inflammation; 2, minimal rash with slight hair loss or inflammation; 3, moderate rash with increased hair loss and mild inflammation; 4, pronounced rash with extensive hair loss and noticeable inflammation; 5, rash extending to the face; 6, presence of a visible wound above the nose. **d** A significant reduction in alopecia and red rash was observed for ETI41 or ETI60 compared with the positive control HCQ or vehicle in SLE-prone mice. **e** Relative lymph node weight compared with body weight. **f**–**h** Serum complement C3 (**f**), antinuclear antibody (**g**) and anti-dsDNA (**h**) levels were measured using ELISA from serum samples. **i** Total IgG content was determined using immunoblotting analysis of kidney samples. Statistical differences between the induced case and other cases were analyzed and verified using a one-tailed Student’s *t*-test (*P < 0.05, **P < 0.01, ***P < 0.001).

SLE pathogenesis is linked to the upregulation of endosomal TLRs, particularly TLR7 and TLR9^39^. We examined the therapeutic potential of ETI41 and ETI60 in a 14-week-old female MRL/lpr mouse model of SLE. ETI41 and ETI60 were orally administered at 30 mg/kg, with hydroxychloroquine (HCQ) as a positive control at 60 mg/kg daily for 39 days (Fig. 5a). Both compounds prevented weight gain or loss throughout the treatment (Fig. 5b). Treatment with ETI41 and ETI60 substantially reduced alopecia and skin rashes compared with the vehicle and HCQ (Fig. 5c,d). Lymph node weights were notably lower, especially in the ETI60 group (Fig. 5e). ETI60 treatment increased complement C3 levels, indicating reduced disease activity and restored immune function (Fig. 5f). Serological markers associated with SLE, such as antinuclear antibody (ANA), anti-dsDNA antibodies and IgG in the kidney, were reduced by ETI41 or ETI60 treatment compared with HCQ treatment (Fig. 5g–i). Despite being administered at half the concentration of HCQ, ETI41 and ETI60 demonstrated a significant reduction in SLE symptoms, suggesting high efficacy at lower doses. Toxicity tests at doses ranging from 150 to 300 mg/kg confirmed their safety (Supplementary Fig. 7). These data indicate the substantial curative potential of ETI41 and ETI60 in SLE.

## Discussion

TLRs activate host immune responses to PAMPs and DAMPs, providing essential defense mechanisms against bacteria, viruses, fungi and parasites^1^. Excessive TLR activation can lead to autoimmune and inflammatory diseases. Endosomal TLRs (TLR3, TLR7, TLR8 and TLR9) have been implicated in the pathogenesis of autoimmune diseases, such as psoriasis, and SLE^17,18^. Although several small-molecule inhibitors targeting endosomal TLRs have been identified and tested in preclinical studies, few have progressed to clinical trials, such as enpatoran (M5049) and MHV370^19,20^. This highlights the urgent need to develop effective inhibitors that can reduce disease progression by targeting endosomal TLRs.

In this study, we identified potent inhibitors of endosomal TLRs from our previously lead compound^32^. SAR analysis led to the successful development of ETI41 and ETI60, which exhibited nanomolar IC_50_ values and demonstrated significantly greater potency and specificity. These lead compounds effectively suppressed the secretion of pro-inflammatory cytokines mediated by endosomal TLR3, TLR7, TLR8 and TLR9 in various cell types, including RAW 264.7 macrophages, human Daudi cells and THP-1 monocytes, as well as in primary murine BMDCs. Importantly, ETI41 and ETI60 selectively inhibited endosomal TLR activity without impairing the responses of cell-surface TLRs, highlighting their specificity and therapeutic potential.

Furthermore, ETI41 and ETI60 substantially blocked downstream signaling pathways involving NFκB (p65), MAPKs (p-ERK, p-JNK and p-p38), Iκ-Bα and IRF7. Structurally, endosomal TLRs are highly similar and grouped into one subfamily^34,40^, making the development of target-specific drugs challenging^33^. These TLRs contain two distinct ligand binding sites: Site I is responsible for recognizing synthetic ligands, such as IMQ, CL075, and R848 in TLR7 and TLR8, and the 5′-xCx DNA motif in TLR9^13,15,41^; and Site II binds single-stranded RNA for TLR7 and TLR8, and CpG DNA motifs for TLR9^16^. Our computational analysis demonstrated that ETI41 and ETI60 establish strong interactions with key residues at Site I, consistent with previously reported ligand interactions^13^. This was further validated by SPR and FACS analyses, which confirmed the strong binding affinity of ETI41 and ETI60 to TLR7 and TLR9, particularly in early endosomes, at neutral pH. These results indicate their effectiveness as inhibitors under physiological conditions.

Excessive activation of endosomal TLRs promotes the overexpression of NFκB–MAPK signaling, exacerbating skin inflammation similar to that observed in psoriasis and SLE^32,42^. We conducted RNA sequencing analysis, which showed that ETI41 and ETI60 modulated the expression of genes associated with immune signaling pathways, particularly those involved in inflammatory responses. In autoimmune diseases, endosomal TLR activation in pDCs plays a crucial role in disease progression by driving excessive immune responses. In SLE, TLR-mediated activation of pDCs leads to aberrant type I IFN production, promoting autoantibody generation, excessive T and B cell activation and immune cell infiltration into target organs^43^. In psoriasis, TLR-driven IL-23 secretion enhances Th17 differentiation and IL-17 production, leading to sustained inflammatory cytokine release and keratinocyte hyperproliferation^44^. ETI inhibitors exert therapeutic effects by modulating type I IFN signaling in pDCs, thereby preventing excessive immune activation and tissue damage in SLE. In addition, ETIs suppress NF-κB signaling, reducing overall immune hyperresponsiveness, and downregulate IL-23 expression, thereby limiting Th17-driven inflammation in psoriasis. These mechanisms highlight ETIs as promising therapeutic candidates for autoimmune diseases driven by dysregulated TLR signaling.

The role of endosomal TLRs extends beyond psoriasis and SLE, encompassing a broader spectrum of diseases, such as arthritis, sepsis and metabolic dysfunction-associated steatohepatitis^45–47^. TLR9 inhibitors can reduce heart failure^48^. In our ongoing research (unpublished data), we discovered that endosomal TLR antagonist 53, a TLR9-specific inhibitor, significantly reduced inflammation and ameliorated myocardial infarction. Our in vivo studies demonstrated the therapeutic efficacy of ETI41 and ETI60 in animal models of psoriasis and SLE. These compounds significantly improved symptoms in IMQ- and IL-23-induced psoriasis models by reducing disease severity and suppressing inflammatory markers, including CD68, IL-23, IL-17A and Ki-67. In addition, ETI41 and ETI60 markedly lowered the levels of key serological markers of SLE, such as ANA, anti-dsDNA and IgG. Collectively, these findings highlight ETI41 and ETI60 as broad-spectrum endosomal TLR inhibitors with strong therapeutic potential for the treatment of autoimmune and inflammatory diseases.

Future research should focus on elucidating the molecular mechanisms underlying the selective inhibition of endosomal TLRs by ETI41 and ETI60. In addition, our data suggest that they do not completely abolish TLR signaling but rather modulate it. Selective inhibition of overactivated TLRs may be beneficial in conditions where dysregulated TLR signaling contributes to disease pathology. Furthermore, exploring the potential synergistic effects of these inhibitors with existing therapeutic agents may provide new avenues for the development of combination therapies for autoimmune diseases. Successful translation of these findings from animal models to human clinical trials is crucial for establishing the clinical efficacy and safety of ETI41 and ETI60. Given the broad involvement of endosomal TLRs in various inflammatory conditions, the development of targeted TLR inhibitors holds significant promise for advancing the treatment of autoimmune and inflammatory diseases.

## Data and materials availability

The RNA-seq data used in the present study are available through the GEO database under accession number GSE255890. All other supporting data of this study are available in the Article and its Supplementary Information. Additional data can be provided by the corresponding author upon reasonable request.

## Supplementary information

Supplementary Information
