# Supplementary material for: Discovery of ETI41 and ETI60: novel selective endosomal Toll-like receptor inhibitors for the treatment of autoimmune diseases
Source: Exp Mol Med. 2025 Sep 1;57(9):1951–62. doi: 10.1038/s12276-025-01526-w (PMC12508057; doi:10.1038/s12276-025-01526-w)
Supplement: Supplementary file 1 — Supplementary Information [file 12276_2025_1526_MOESM1_ESM.pdf]

Supplementary information

Discovery of ETI41 and ETI60: Novel Selective Endosomal Toll-Like Receptor Inhibitors for the Treatment of Autoimmune Diseases

Uisuk Jeong[a,b,1], Wang Hee Lee[b,1], Yang Seon Choi[b,1], Muhammad Haseeb[a], Wook-Young Baek[b], Ji Hye Han[b], Hongjoon Choi[a,b], Moon Suk Kim[b], Chang-Hee Suh[b], Wook Kim[b,c,*], Sangdun Choi[a,b,c,*]

[a] S&K Therapeutics, Ajou University Campus Plaza 418, Worldcup-ro 199, Yeongton-gu, Suwon 16502, Korea

[b] Department of Molecular Science and Technology, Ajou University, Suwon 16499, Korea

[c] Advanced College of Bio-convergence Engineering, Ajou University, Suwon 16499, Korea

[1] These authors contributed equally to this work

* Corresponding authors:

Wook Kim: wookkim21@ajou.ac.kr

Sangdun Choi: sangdunchoi@ajou.ac.kr

This file includes:

1. Synthesis procedures
2. Supplementary Figures. 1 to 7

< Synthesis Procedures >

General procedure for preparation of compound ETI41

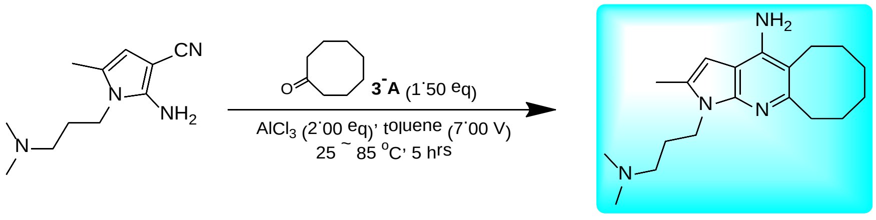

3 ETI41

To a suspension of compound 3-A (1.84 g, 14.5 mmol, 1.50 eq), and $AlCl_3$ (2.59 g, 19.3 mmol, 2.00 eq) in toluene (14.0 mL, 7.0 V) were added to compound 3 (2.00 g, 9.70 mmol, and 1.00 eq) and added to 25 °C under N_2. After addition, the mixture was stirred at 85 °C for 5 h. Liquid chromatography–mass spectrometry (LC-MS) (ET51512-3-P1C1, product: Retention Time (RT) = 1.55 min) revealed that the starting material had been consumed. H_2O (20.0 mL, 10.0 V) and dichloromethane (20.0 mL, 10.0 V) were added and separated. The organic phase was dried over anhydrous Na_2SO_4, filtered, and concentrated under vacuum to obtain a residue, which was purified by reversed-phase High-performance liquid chromatography (HPLC) [water (NH_3H_2O + NH_4HCO_3) - ACN; B%: 15% - 65%, 8 min] to obtain the desired product ETI41 [1.10 g, 36.0% yield, proton nuclear magnetic resonance (1H NMR) ET51512-3-P1D1] as a brown solid.

General procedure for preparation of ETI60

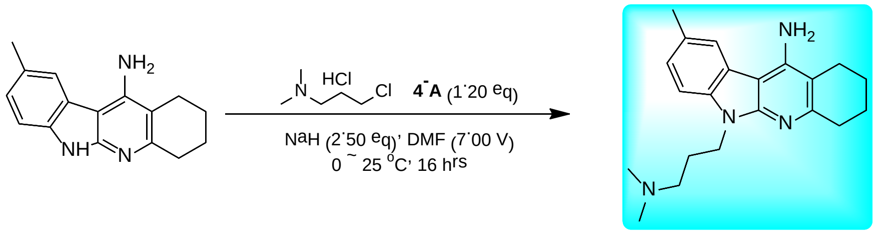

4

ETI60

To a solution of compound 4 (4.00 g, 15.9 mmol, 1.00 eq) in Dimethylformamide (28.0 mL, 7.00 V) was added NaH (1.59 g, 39.7 mmol, 2.50 eq) at 0 °C under N_2. Then, compound 4-A (3.02 g, 19.1 mmol, 1.20 eq) was added to the mixture at 25 °C. After addition, the reaction mixture was stirred at 25 °C for 16 h. LC-MS (ET51512-8-P1C1, product: RT = 1.65 min) revealed that the starting material had been consumed. The mixture was then added to H_2O (8.00 mL, 2.00 V) and EtOAc (80.0, 20.0 V) and separated. The organic phase was dried over anhydrous Na_2SO_4, filtered, and concentrated under vacuum to yield a residue, which was purified by reverse-phase HPLC [water (NH_3H_2O + NH_4HCO_3) - ACN; B%: 15% - 65%, 8 min] to obtain the desired product ETI60 (1.20 g, 22.3 % yield) as an off-white solid, which was confirmed by 1H NMR (ET51512-8-P1D1).

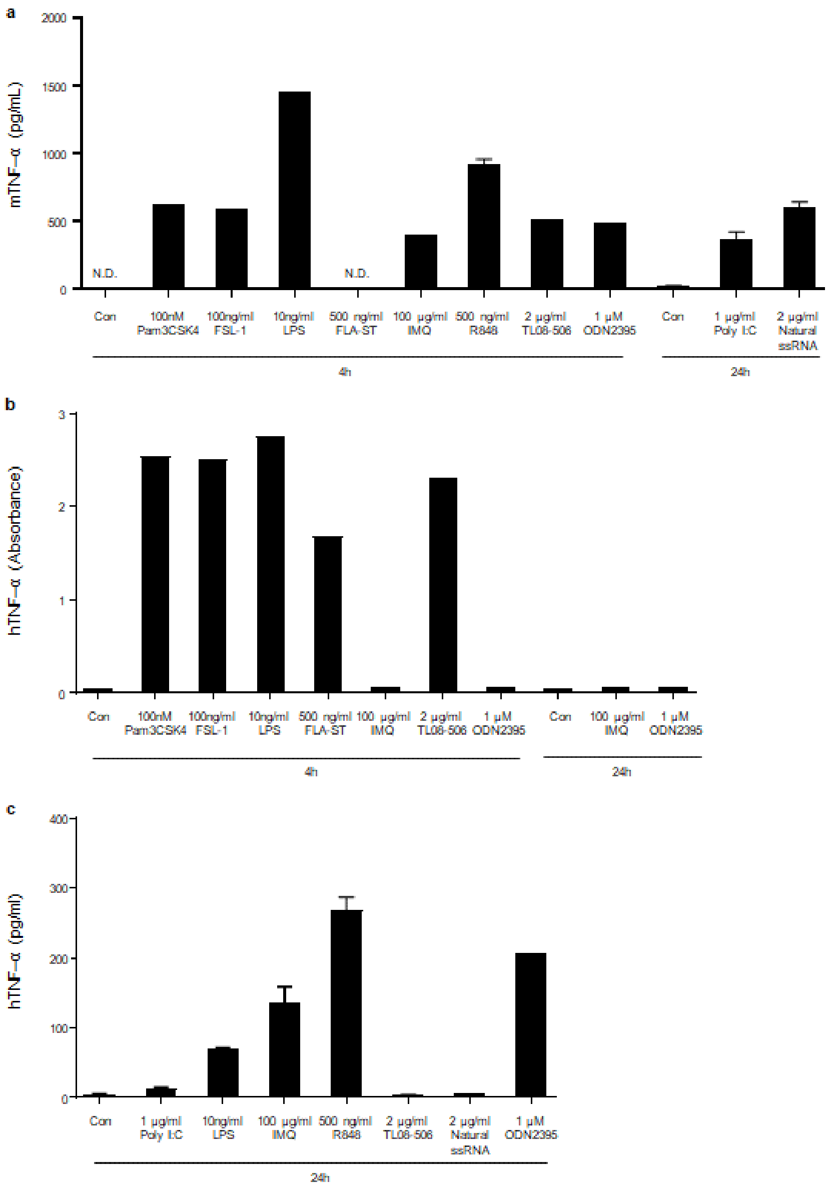

Supplementary Fig. 1. The ligand study optimization for each TLR. TNF-α secretion was quantified and responses to each TLR were assessed using ELISA in mouse and human cell lines. The cells were activated with agonistic ligands: TLR1/2 (FSL-1, 100 ng/mL, 4 h), TLR2/6 (Pam3CSK4, 100 nM, 4 h), TLR3 (poly I:C, 1 μg/mL, 24 h), TLR4 (LPS, 10 ng/mL, 4h), TLR5 (FLA-ST, 500 ng/mL, 4 h), TLR7 (ORN06/LyoVec, 2 μg/mL, 24 h; and Imiquimod, 1 μg/mL, 4h or 24h), TLR8 (TL8-506, 2 μg/mL, 24 h), and TLR9 (ODN2395, 1 μM, 4 h or 24h). (**a**) In the murine Raw264.7 cell line, mTNF secretion was measured in response to TLR 1, 2, 3, 4, 5, 6, 7, 8, 9. (**b**) In the human THP-1 cell line, hTNF secretion was measured in response to TLR 1, 2, 3, 4, 5, 6, 7, 8, 9. (**c**) In the human Daudi cell line, hTNF secretion was measured in response to TLR 3, 4, 7, 8, 9.

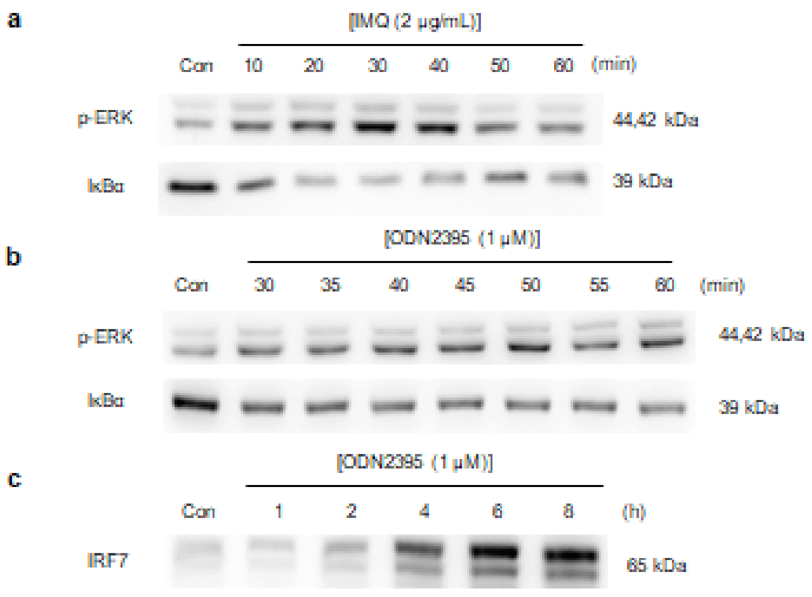

Supplementary Fig. 2. Time-dependent Western blotting optimization in TLR7 and TLR9. Cytoplasmic markers p-ERK, IκBα, and IRF7 were selected as indicators of intracellular changes in RAW264.7 cells. (**a**) IMQ (2 μg/ml) was used to track changes in cytoplasmic markers at 10-minute intervals for up to 1 hour. (**b**) ODN2395 (1 μM) was used to track changes at 5-minute intervals from 30 minutes to 60 minutes. (**c**) The expression of IRF7 following ODN2395 treatment was monitored at 1-hour intervals for up to 2 hours, and then at 2-hour intervals for up to 8 hours.

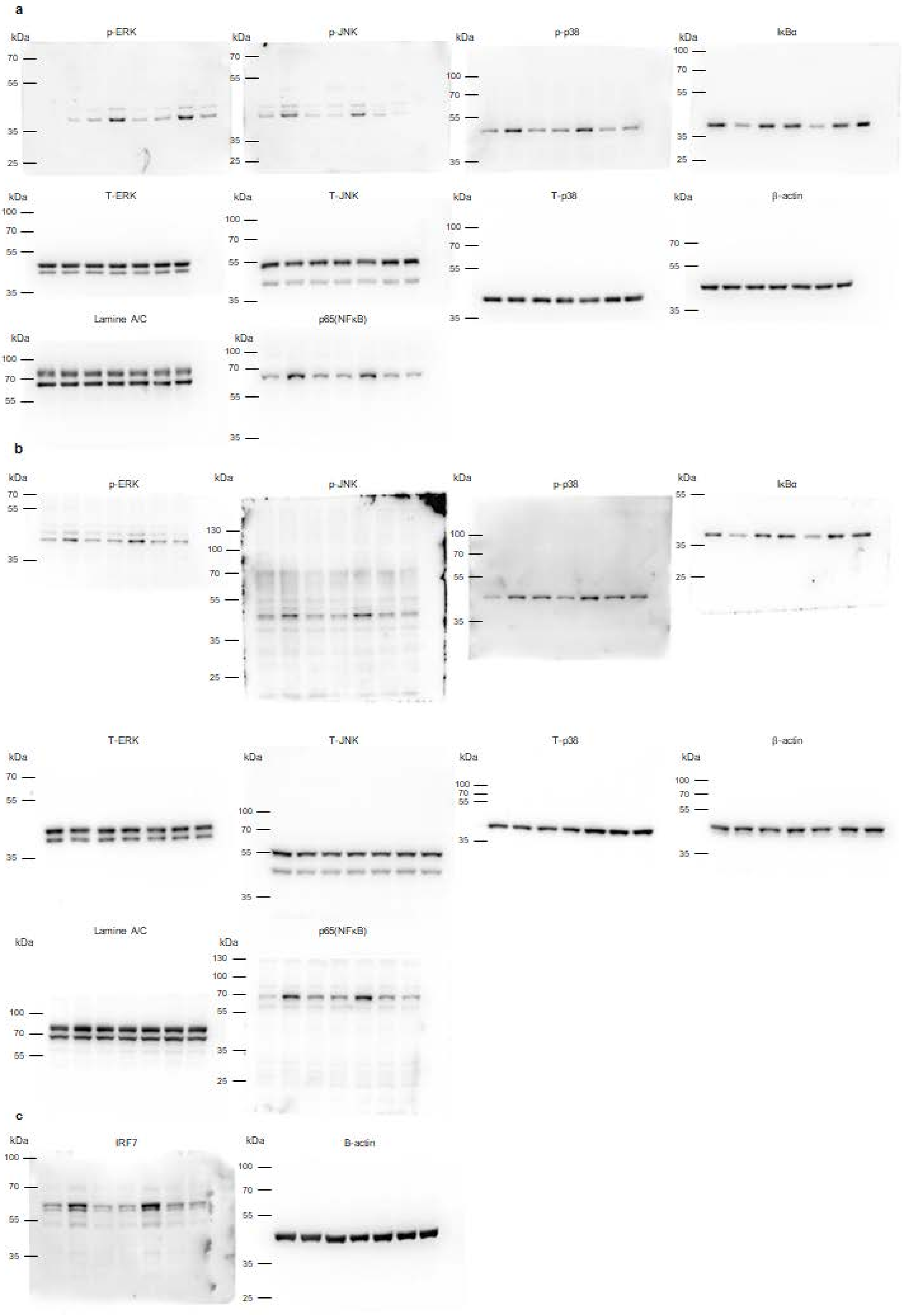

Supplementary Fig. 3. Raw images of western blotting. (**a**) Un-cropped images for Fig. 2a. (**b**) Un-cropped images for Fig. 2b. (**c**) Un-cropped images for Fig. 2c. The labeling of the bands on all membranes is identical to the order indicated in Fig 2a-c.

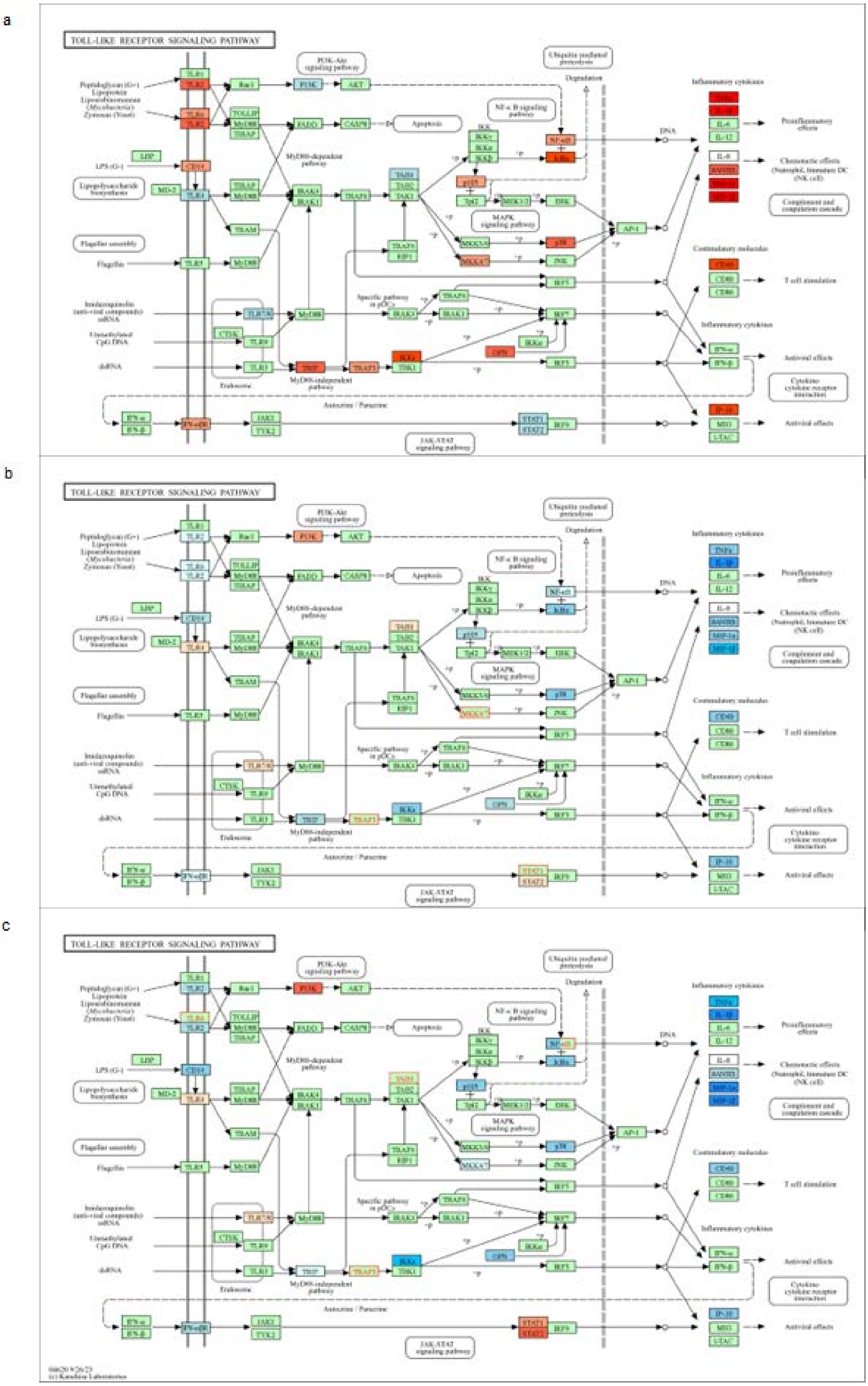
a
b
c
TOLL-LIKE RECEPTOR SIGNALING PATHWAY

Supplementary Fig. 4. Transcriptomic Analysis of TLR downstream signaling in RAW 264.7 cells. RNA-seq analysis was performed to investigate TLR7-induced inflammatory gene expression regulated by ETI41 or ETI60. (**a**) Changes in mRNA expression patterns observed after treatment for 2 h with IMQ (1 μg/mL) compared to the untreated control. (**b**) Changes in mRNA patterns observed after pre-treatment with ETI41 (1 μM) for 30 min, followed by IMQ treatment for 2 h. (**c**) Changes in mRNA patterns observed after pre-treatment with ETI60 (1 μM) for 30 min, followed by IMQ treatment for 2 h. Blue indicates down-regulation of genes, while red indicates up-regulation.

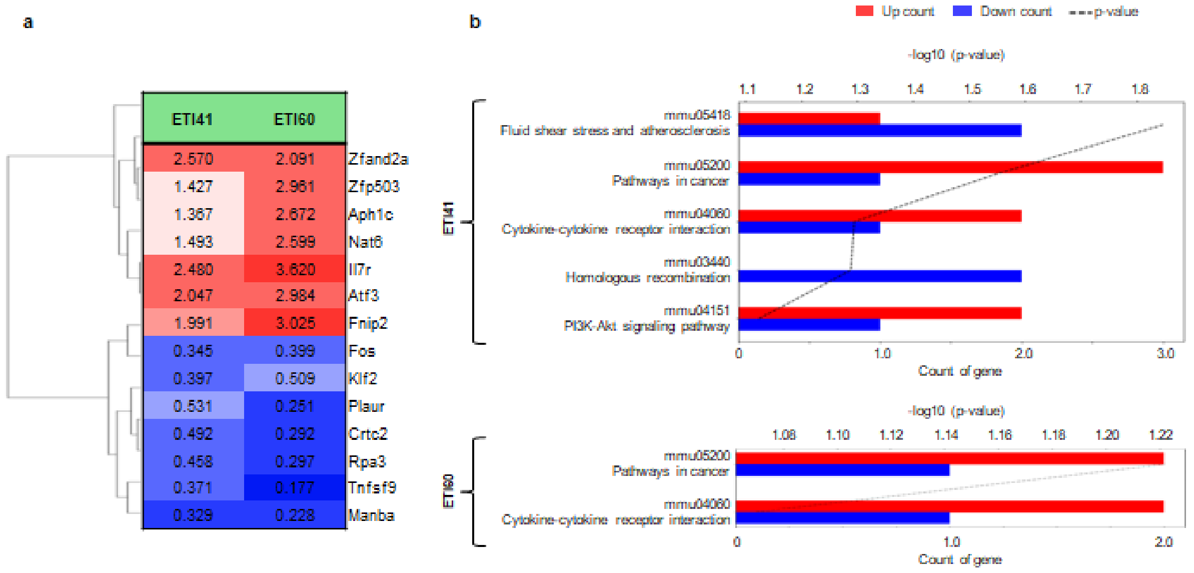

Supplementary Fig. 5. Potential off-target effects of ETI41 and ETI60. ETI41 and ETI60 were administered at a concentration of 10 μM for 2.5 hours in RAW 264.7 cells. (**a**) Among the 23,282 genes analyzed, RNA-seq identified significant expression changes (fold change > 2.5, p-value < 0.05) when ETI41 or ETI60 was treated alone without IMQ were represented as a heatmap. (**b**) Pathways associated with significantly altered gene expression (fold change > 2.0, p-value < 0.05) were classified using KEGG. Fold-change values relative to the non-induced condition are shown, with pathways enriched in upregulated genes indicated in red and those enriched in downregulated genes indicated in blue.

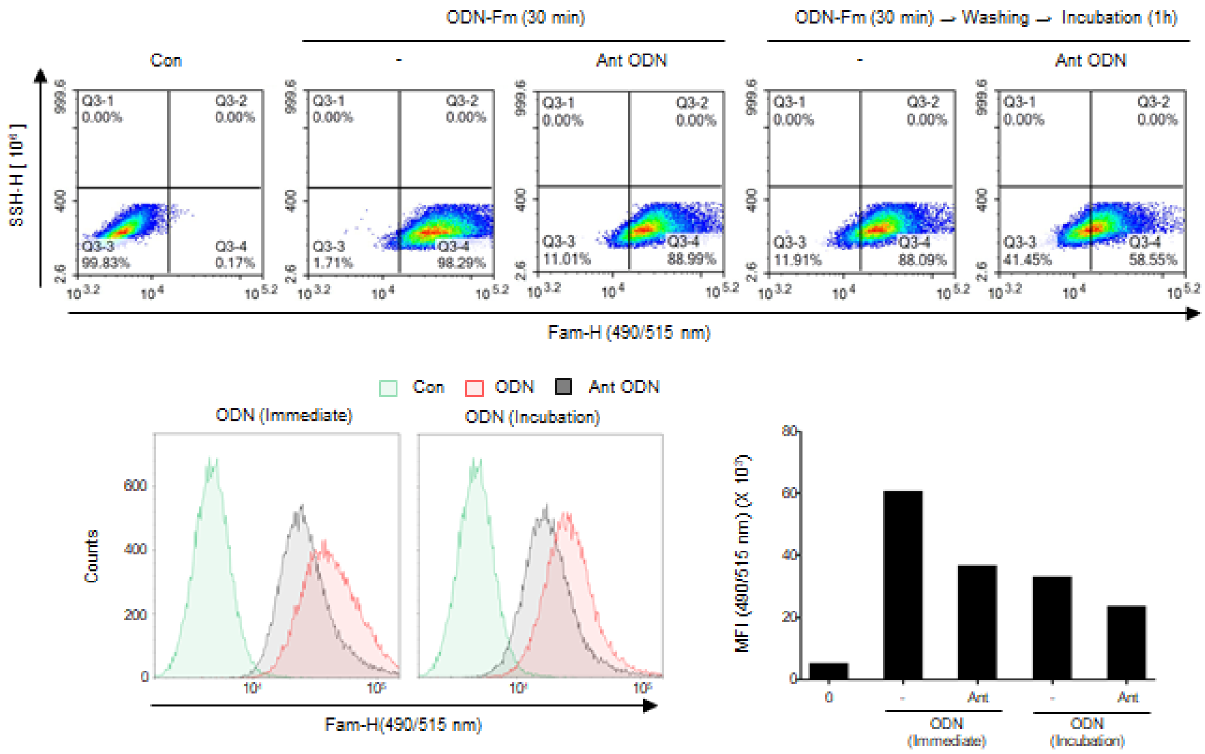

Supplementary Fig. 6. Antagonist ODN competitively blocks agonistic ODN in both early and late stages of endosomes. RAW 264.7 cells were treated with antagonist ODN (1 μM) prior to the ODN-FAM reaction. After 30 min of stimulation, the cells were harvested and analyzed with or without a 1 h incubation process.

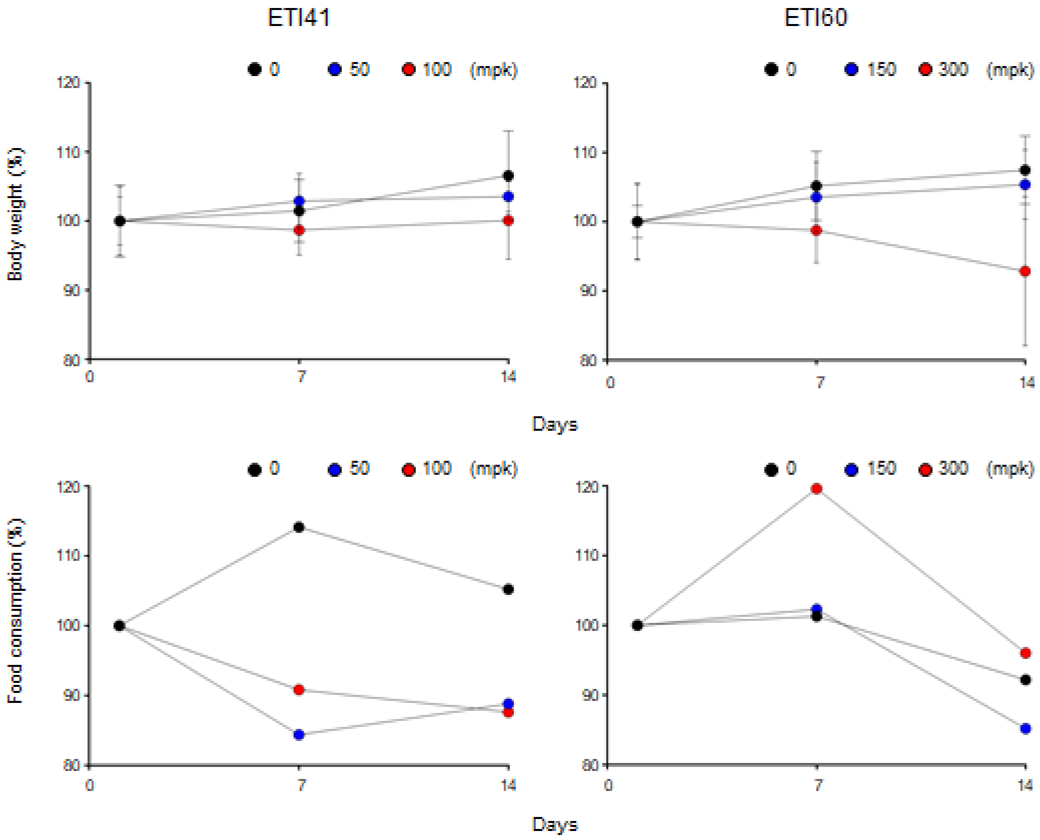

Supplementary Fig. 7. Animal lethality test of high-dose ETI series. The ETI series was administered daily *via* oral route for 14 days. Changes in relative body weight and food consumption were monitored at the beginning, on day 7, and on day 14.
